# Investigation of the clinical performance of a novel solid‐state diagnostic dosimeter

**DOI:** 10.1120/jacmp.v16i4.5439

**Published:** 2015-07-08

**Authors:** Jason Tse, Donald McLean

**Affiliations:** ^1^ Medical Physics and Radiation Engineering Canberra Hospital Garran ACT 2605 Australia

**Keywords:** diagnostic dosimeter, solid‐state dosimeter, fluoroscopic dosimetry, fluoroscopy system

## Abstract

This study investigated the clinical performance of a novel solid‐state diagnostic dosimeter, the RaySafe Xi transparent detector, by comparing its performance to a reference‐class ionization chamber. Firstly a comparison of dosimeter response “free‐in‐air” with standard beam qualities was made, followed by an investigation into its relative transparency in an X‐ray field and angular sensitivity dependence. The second part of the study looked at the overall performance of the transparent detector under scatter conditions with a number of beam qualities, including standard beam and those hardened by copper (Cu) filtration of thickness up to 0.9 mm, as would be encountered in the equipment testing of fluoroscopy systems. Overall, the transparent detector has demonstrated equivalent measurement properties to the ionization chamber under standard conditions and provided similar X‐ray attenuation as reflected by the nearly identical radiographic parameters selected for both dosimeters by the automatic dose rate control (ADRC) system. Yet, it also possessed an asymmetric angular response which respectively under‐ and overestimated the dose contribution from the rear and lateral directions by the same amount of 50%. The transparent detector provided comparable dose reading of ±3% to the ionization chamber with standard beam qualities and backscatter radiation present. These results were in good agreement with those of free‐in‐air measurement, indicating that the angular under‐ and overresponse might potentially compensate one another for accurate measurement. However, for identical Cu filtered beam qualities and setups, the transparent detector on average overresponded by 5.4% across the useful tube voltage range. In conclusion, the transparent detector, with its novel design, is essentially equivalent, within a 5% tolerance, to an ionization chamber, except in situations where beams hardened with Cu filtration are used with backscatter radiation present requiring larger uncertainty error estimations.

PACS numbers: 87.57 uq, 87.59.C‐, 87.59.Dj

## I. INTRODUCTION

Solid‐state dosimeters have an increased utilization in quality assurance (QA) testing of diagnostic X‐ray systems due to significant advances in dosimeter design in an environment with increased radiation safety requirements. One significant advantage of these dosimeters over traditional ionization chambers is the convenience of measurement that can be provided. From a single irradiation, they are able to determine the air kerma, tube voltage, half value layer (HVL), and exposure time, as well as the output waveform. Yet, end‐users normally have limited access to the in‐depth knowledge of the dosimeters' design and operation which is directly related to the measurement limitations of the devices. Ultimately, this incomplete understanding of the dosimeters' limitations can constitute a major source of systematic errors in dosimetry measurement, especially in clinical beam conditions.

In the medical physics community, it is generally agreed that diagnostic dosimeters ought to comply with the International Electrotechnical Commission (IEC) standard 61674.^(1)^ However, it is noted that the IEC standard does not include testing in the clinical conditions of incident primary X‐ray field with associated scatter conditions as experienced in entrance air kerma measurement.^(2)^ Moreover, the standard only addresses dosimetry measurements with standard beam qualities, such as the RQR and RQA series, which are not necessarily representative of the ones used clinically.^3^, ^4^ For instance, all the RQR and RQA beams utilize aluminum (Al) filtration, while modern fluoroscopy and interventional systems commonly incorporate copper (Cu) filtrations (with thickness going up to 0.9 mm) for better management of patient dose. Apart from Cu, other materials with high atomic number, such as gadolinium (Gd), gold (Au), and tantalum (Ta), have also been used in certain commercial systems to offer more aggressive filtration.^(3)^ As a result of applying these additional filters, the X‐ray energy spectrum will be entirely different to those of the standard beams, even with similar values of HVL.

The RaySafe Xi transparent detector (RaySafe AB, Billdal, Sweden) indicates in its promotional material that it is ‘transparent’ in the X‐ray field, dedicated for dosimetry measurements of X‐ray systems operated with automatic dose rate control (ADRC). Examples of such systems include fluoroscopy and interventional X‐ray systems with automatic feedback circuits to ‘control’ the radiation dose rate by real‐time adjustment of the acquisition parameters in response to the attenuation of the X‐ray field.^(4)^ As stated by the vendor, this solid‐state dosimeter is specially designed to measure entrance air kerma rate, which is an essential QA measurement for fluoroscopy X‐ray systems. Also indicated is the range of operation dose rate from 100 nGy/s to 20 mGy/s, confirming the dosimeter's suitability for fluoroscopic dosimetry.

It is the aim of this study to investigate the properties of the transparency detector including its clinical performance characteristics as compared to a reference ionization chamber, and its suitability for implementation into routine QA practice.

## II. MATERIALS AND METHODS

### A. Instrumentation and experimental setup

The RaySafe Xi transparent detector is a novel solid‐state dosimeter and was radiographed to determine the physical components of the detector. The ionization chamber used as a comparison device in this study was manufactured by Radcal (Model: 10X6‐6; Radcal Corporation, Monrovia, CA). It is a general purpose ionization chamber with dose and dose‐rate range useful for dosimetry measurements of radiography and fluoroscopy systems. This chamber is cylindrical with an active volume of 6 cm^3^. Owing to its symmetrical configuration, dosimeters of this kind exhibit a uniform response with the incident angle of radiation.^5^, ^6^ Moreover, the response of ionization chambers is generally energy independent of X‐ray energies as a result of its mode of radiation detection (i.e., gaseous ionization) and the chamber wall material of polycarbonate. According to the vendor, the 10X series chambers respond within ±5% across the energy range from 30 keV to 1.33 MeV. Additionally, such detectors can be considered as transparent to X‐ray radiation. These two criteria have made them the conventional choices for dosimetry measurements of fluoroscopy and interventional systems.

From communications with the vendors (J. Tse, personal communication, 05/15/2013 and 04/01/2014), both the 10X6‐6 ionization chamber and transparent detector are in compliance with the IEC standard 61674 for applicable sections. Both dosimeters were under the maintenance programs by the vendors with their calibrations traceable to National Institute of Standards and Technology (NIST). The calibrations were conducted with a range of RQR beam qualities. A summary of the dosimeters' technical specifications is provided in Table 1

**Figure 1:**
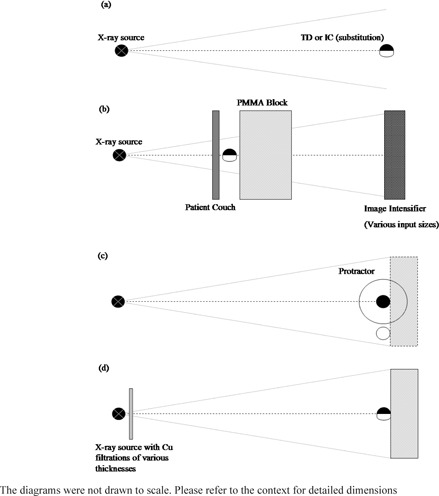
Illustrations of the experimental setup for (a) free‐in‐air comparison, (b) dosimeters' transparency investigation, (c) angular dependence of the transparent detector, and (d) the overall performance of dosimeters under clinical measurement conditions. In the figures, the transparent detector and the ionization chamber were represented by • and ○, respectively.

A number of experimental setups were used in the study, as illustrated in Fig. 1. With the exception of the investigation of angular dependence, all measurements were made using the substitution method: alternative air‐kerma measurements were made with the different dosimeters along the central axis of the beam at the same distance from the focal spot. For all tests, each measurement was repeated three times to improve the precision of the results, and the influence of the heel effect was minimized by orienting the dosimeters perpendicular to the cathode–anode axis. The hospital beam qualities used in the experiments are detailed in 2, 3. Each beam quality was assigned a specific beam ID depicting the center name, The Canberra Hospital (TCH), followed by the tube voltage (kVp) and the thickness of Cu filtration if applicable.

**Table 1:** Technical specifications of the dosimeters used in the study

*Manufacturer*	*Model*	*Type*	*Dose Range*	*Dose Rate Range*	*Energy Dependence*
Raysafe	Transparent	SD	10 nGy−9999 Gy	100 nGy/s−20 mGy/s	± 5% 60–150 kVp, HVL: 2–10 mmAl^a^
Radcal	10X6−6	IC	100 nGy−516 Gy	20 nGy/s–149 mGy/s	± 5% 30 keV to 1.33 MeV

a ^a^ 13 mm Al added filtration at 145 kVp gives a HVL of ∼10 mm Al.

IC=ionization chamber; SD=solid‐state detector.

**Table 2:** The hospital beam qualities used in in‐air comparison of dosimeter's response and their standard beam qualities counterparts

*Standard Beam Qualities for Calibration*			*Hospital Beam Qualities*			
*Beam Qualities*	*kVp*	*HVL (mm Al)*	*Beam ID*	*kVp*	*Total Filtration*	*HVL (mm Al)*
RQR 3	50	1.78	TCH 50	50	2.5 mm Al	1.91
RQR 5	70	2.58	TCH 70	70	2.5 mm Al	2.61
RQR 8	100	3.97	TCH 102	102	2.5 mm Al	3.86

**Table 3:** The hospital beam qualities used in the evaluations of dosimeters' performance under clinical measurement conditions

*Beam ID*	*Filtration*	*kVp Range*	*HVL Range (mm Al)*
TCH kVp^a^	2.5 mm Al (inherent)	50–125	1.91–4.69
TCH kVp Cu0.4	2.5 mm Al (inherent)+0.4 mm Cu	50–125	4.16–9.45
TCH kVp Cu0.6	2.5 mm Al (inherent)+0.6 mm Cu	50–125	4.65–10.35
TCH kVp Cu0.9	2.5 mm Al (inherent)+0.9 mm Cu	50–125	5.13–11.26

a ^a^ kVp refers to tube voltage settings of 50, 60, 70, 81, 102, 125. Please refer to text for details of naming convention.

### B. In‐air comparison

Radiation exposures were performed with a fixed radiographic system (Fig. 1(a)) using hospital beam qualities, TCH_50, TCH_70, and TCH_102, which closely simulated the standard RQR beam qualities, RQR 3, 5, and 8 (Table 2). Prior to use, the system had passed routine quality assurance (QA) tests, including tube voltage accuracy, reproducibility, and output linearity, based on the acceptance criteria from Environment Protection Authority (EPA) in New South Wales, Australia.^(7)^ The measurements were made at a distance of 1500 mm from the focal spot to minimize the effect of X‐ray field nonuniformity.

### C. Detector relative transparency in ADRC X‐ray fields

The transparent and comparison dosimeters were sequentially irradiated in conjunction with 200 mm PMMA attenuation material using a fixed fluoroscopy systems operating under ADRC (Fig. 1(b)). The system had also passed the relevant equipment QA test criteria from EPA.^(7)^ The ADRC mode selected was endoscopic retrograde cholangiopancreatography (ERCP) and the pulse rate was fixed at 10 pulses per second. The focal spot to image intensifier distance was fixed at 1000 mm and the dosimeter was positioned at the central region of the field on the tube side of the attenuator and at a distance of 300 mm from the surface of the image intensifier. The resultant acquisition parameters, including kVp, tube current (mA), added filtration (mm Cu), and pulse width (s) were recorded and compared for each dosimeter.

### D. Angular dependence

The complete angular sensitivity dependence over 360° was studied by irradiating the transparent detector to beam TCH_81 at different orientation angles measured using the protractor with an uncertainty of ± 0.5° (Fig. 1(c)). Clockwise rotation of the detector was regarded as positive and the reverse was taken as negative. The study was conducted with and without the underlying Solid Water phantom (Gammex, Middleton, WI) of thickness 200 mm to investigate any influence of backscatter radiation on the angular sensitivity dependence. Changes in sensitivity due to small angle variations of 5° increment were also investigated. To minimize the influence caused by output fluctuation of the X‐ray machine, an ionization chamber was used as a monitor chamber at a position not affecting the reading of the transparent detector. All the readings were normalized to the reading at the recommended incidence orientation. Subsequently, the experiment was repeated with two other beam qualities TCH_60 and TCH_102.

### E. Overall performance in clinical conditions

The clinical performances of the dosimeters were ultimately compared under clinical conditions that included hardened beams and backscatter radiation. Irradiations were performed with the fixed radiographic system (Fig. 1(d)) using tube voltages of 50, 60, 70, 81, 102, and 125 kVp with only with the inherent Al filtration of 2.5 mm (TCH_kVp beams in Table 3) followed by added Cu filters of thickness 0.4 mm, 0.6 mm and 0.9 mm at the X‐ray tube exit (TCH_kVp_Cu beams in Table 3). To generate the backscatter radiation, a Solid Water phantom of 200 mm was positioned immediately behind the dosimeters for irradiations.

## III. RESULTS

### A. Instrumentation details

**Figure 2:**
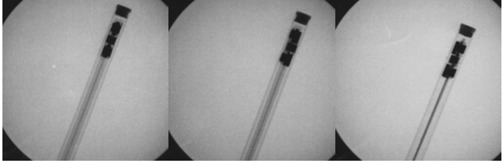
Radiographic images of the transparent detector at 0° (left), 45° (middle), and 90° (right) towards the primary radiation field.

Radiographic images of this dosimeter (Fig. 2) showed that there was no lead backing present, making the device essentially transparent in the X‐ray field and able to detect radiation incident from all angles. It was also revealed that the sensitive volume of the dosimeter contained separate groups of sensor elements facing in orthogonal directions. This specific configuration was believed to account for the energy dependence, as well as the angular sensitivity dependence. The sensitive area and recommended orientation of the transparent detector to an incident X‐ray field is indicated by a white rectangle near to the detector tip. It is recommended by the manufacturer that the sensitive area should face directly towards the radiation source for the most accurate readings.

### B. In‐air comparison

Irradiated free‐in‐air with three RQR beam qualities, the responses of the two dosimeters agreed within 3%, thereby reflecting their equivalence of performance under standard measurement conditions. These results were expected for both detectors were in compliance with the IEC standard 61674.^(1)^

### C. Detector relative transparency in ADRC controlled X‐ray fields

The ADRC acquisition factors and the displayed dose rates as a result of positioning the transparent detector or the ionization chamber in the beam were largely identical, indicating that both dosimeters exhibited the same small amount of minimal attenuation to be ‘seen’ by the ADRC feedback circuit (Table 4).

**Table 4:** The output of the ADRC as a result of positioning the transparent detector and the ionization chamber in the radiation field. The bolded and italic figures represent the results of the transparent detector (number before the slash) and the ionization chamber (number after the slash), respectively

*ADRC Mode*	*Endoscopic Retrograde Cholangiopancreatography (ERCP)*				
			*ADRC Output*		
*Field Size (cm)*	*kVp*	*mA*	*Added Filter (mm Cu)*	*Pulse Width (ms)*	*Displayed Dose Rate (mGy/min)*
14	104.2 / 107	31 / 30.5	0.2 / 0.2	10.2 / 10.2	23 / 23
20	83.2 / 84.7	38.4 / 37.5	0.2 / 0.2	10.1 / 10.2	13 / 14
28	70 / 70	27 / 28	0.2 / 0.2	3.3 / 3.6	2 / 2
40	70 / 71	47 / 46	0.6 / 0.6	11 / 11.3	3 / 3

### D. Angular sensitivity dependence

**Figure 3:**
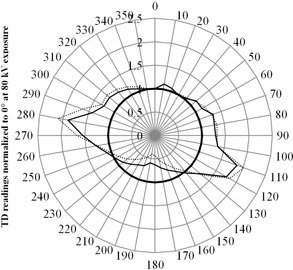
Normalized angular sensitivity dependence of the transparent detector at 81 kVp exposure for mixed field (—), for in‐air measurements (—), and for the ideal response with no angular sensitivity dependence (—).

The angular sensitivity of the transparent detector at 81 kVp was asymmetrical, as seen in Fig. 3, with little dependence on the presence of secondary scatter. For both sets of data, there are significant overresponses of over 50% from 270° to 290° and from 100° to 130°. Underresponses generally occurred from 140° to 240°, and the largest underresponses of about 50% were observed from 180° to 190°. The angular sensitivity dependence measured at both 60 kVp and 102 kVp showed a very similar distribution.

**Figure 4:**
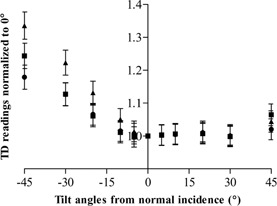
Angular sensitivity dependence of the TD for small tilt angles at 60 kVp (▴), 81 kVp (▪), and 100 kVp (•) exposures.

For small tilt angles (i.e., ± 5° and ± 10°), the response was within ±3% for the three kVp settings (Fig. 4). For positively tilted angles, the dosimeter did not have a significant overresponse before +30° as compared to the more rapid increment of response for negatively tilted angles, as seen at −30° with the dosimeter overresponded by over 10% for 60 kVp and 81 kVp and 20% for 102 kVp.

### E. Overall performance in scatter conditions

**Figure 5:**
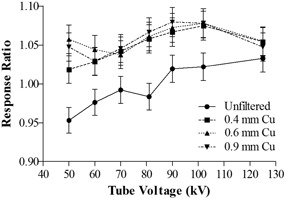
Relative response of the transparent detector to the ionization under various filtered beam qualities and the presence of backscatter radiation

In Fig. 5, the two dosimeters produced results comparable within ±5% for the full range of unfiltered beam qualities. By omitting the reading of 50 kVp (beyond the specification of the transparent detector), the agreement could be improved to ±3% which was consistent with the results of in‐air measurements, implying that the behavior of the transparent detector did not vary with the introduction of the backscatter radiation under normal beam qualities. It was also noted that the response ratio generally increased with the value of kVp as a result of a limited degree of energy dependence by the transparent detector.

Concerning other hardened beam qualities, the response ratios exhibited similar increasing trends across the range of tube voltages. Compared to the ionization chamber, the transparent detector on average overresponded by approximately 4.89% (coefficient of variance (COV): 1.94%), 5.78% (COV: 1.35%), and 5.61% (COV: 1.78%) for Cu filtrations of thickness 0.4 mm, 0.6 mm, and 0.9 mm, respectively.

## IV. DISCUSSION

The transparent detector had a similar response to the reference ionization chamber under standard conditions. This confirmed the statement by the vendors^(8)^ that its response was generally equivalent to a typical ionization chamber. The vendor‐supplied information on its dose rate limit was also verified experimentally, and it was noted that the dose‐rate response was such that the dosimeter might not be suitable for some radiographic dose rates. When backscatter radiation was present, the relative response of the transparent detector demonstrated a larger degree of variation, but was still within ±5% to that of the ionization chamber for standard beam qualities. However for other beam qualities, particularly those including Cu filtration, the difference between the ionization chamber and the transparent detector's readings increased to an average of 5.4% for tube voltages ranging from 50 to 125 kVp. This difference is thought to be attributed to the energy dependence, which is intrinsic to solid‐state dosimeters.

The additional consideration of backscatter radiation complicated the detector energy dependence due to the additional and different energy spectrum involved. The simulation by Benmakhlouf and colleagues,^(9)^ shows that the resultant effective energy spectrum will be lowered once backscatter is considered. Additionally, it is noted that the HVLs of some of the Cu filtered beams exceeded the upper detection limit of the transparent detector (1, 2).

Angular sensitivity dependence, as with energy dependence, is an intrinsic limitation of solid‐state dosimeters.^(5)^ If not corrected, this could be problematic especially when measuring entrance surface kerma as the backscatter radiation interacts with the detector surface from multiple angles. Conventionally, a lead backing would be mounted at the rear of this kind of dosimeter to avoid the backscatter radiation from compromising the final reading. Subsequently, a suitable backscatter factor would need to be applied to convert the incident surface kerma measured to the entrance surface kerma. The construction of the transparent detector, as described, differentiates itself from its peers by possessing no single lead backing and being able to detect radiation from all angles. Converse to the uniform response of a typical ionization chamber, it has demonstrated a substantially asymmetrical angular dependence over 360°, which could be attributed to its specific arrangement of the detector elements (Fig. 1). Moreover, this angular distribution was independent of beam energy and the inclusion of backscatter radiation. It was postulated by the authors that the asymmetrical response was specially designed for measuring backscatter radiation with the 50% overresponse at the lateral directions (270°–290° and 100°–130°) giving a counter balance to the 50% underresponse at the back of the detector (140°–240°). This postulation was strengthened by the comparable results of the two dosimeters in normal measurement situations (Table 2). Note that when manually orientating the transparent detector as indicated towards the X‐ray source, any small angle tilts which can commonly occur result in a maximum reading uncertainty of ± 3%.

Clinical dosimetry plays an essential part in the QA testing of X‐ray equipment and in the assessment of radiation risk in diagnostic radiology practice. The term ‘clinical’ underscores the important distinction between the measurement conditions in a standard calibration laboratory and those in the clinical setting. Ideally every dosimeter ought to be calibrated with the beam qualities and conditions in clinical use with a measurement uncertainty of ±7% (coverage factor k=2), as recommended by the International Atomic Energy Agency (IAEA).^(10)^ Nevertheless, owing to the high variability in the measurement conditions, including the wide range of primary beam qualities and the involvement of backscatter radiation, this practice is not practical in the context of diagnostic radiology. Hourdakis and colleagues^(2)^ concluded, from their evaluation of dosimeters, that a minimum dosimeter calibration with standard beam qualities RQR 3, RQR 5, and RQR 9 were required to guarantee the accuracy of measurements to be less than 3%, provided that IEC standard 61674 was complied with and account was taken of the energy dependence. Their study, however, did not address beam qualities that were heavily filtered and the dosimeters' response to scatter radiation. In the current situation, in which there is not yet an international consensus for standardizing the clinical beam qualities for dosimeter calibration, additional calibration with RQA beam qualities and a series of in‐house dosimeter evaluation, as described in present study, will be necessary for a more accurate estimate of the measurement uncertainty.

## V. CONCLUSIONS

The transparent detector is one of the few diagnostic solid‐state dosimeters which accommodate the measurement of backscatter radiation. Yet, as mentioned earlier, it is also of critical importance to have an in‐depth understanding of the dosimeter's properties and its associated limitations, to prevent any systematic measurement errors occurring during measurement.

The present study has confirmed that the transparent detector, with its novel design, is essentially equivalent, within a 5% tolerance, to an ionization chamber under standard, or close‐to‐standard, beam qualities. Yet, users should expect a larger measurement error of more than 5% with this dosimeter when the measurement involves Cu‐filtered X‐ray beams. The methodologies in the present study can also be applied to other commercial solid‐state dosimeters to verify their performance under clinical measurement conditions. Ultimately, users should rely on the data of standard calibrations, the dosimeter's specification, and a set of in‐house dosimeters' evaluations to estimate the measurement errors involved.
